# Contusion spinal cord injury upregulates p53 protein expression in rat soleus muscle at multiple timepoints but not key senescence cytokines

**DOI:** 10.14814/phy2.14357

**Published:** 2020-02-05

**Authors:** Zachary A. Graham, Abigail Goldberger, Daniella Azulai, Christine F. Conover, Fan Ye, William A. Bauman, Christopher P. Cardozo, Joshua F. Yarrow

**Affiliations:** ^1^ Research Service Birmingham VA Medical Center Birmingham AL USA; ^2^ Department of Cell, Developmental and Integrative Biology University of Alabama‐Birmingham Birmingham AL USA; ^3^ Center for the Medical Consequences of Spinal Cord Injury James J. Peters VA Medical Center Bronx NY USA; ^4^ Icahn School of Medicine at Mount Sinai New York NY USA; ^5^ Research Service and Brain Rehabilitation Research Center Malcolm Randall VA Medical Center North Florida/South Georgia Veterans Health System Gainesville FL USA; ^6^ Division of Endocrinology, Diabetes, and Metabolism University of Florida College of Medicine Gainesville FL USA

**Keywords:** cytokines, inflammation, paralysis, SASP, senescence, spinal cord injury

## Abstract

To determine whether muscle disuse after a spinal cord injury (SCI) produces elevated markers of cellular senescence and induces markers of the senescence‐associated secretory phenotypes (SASPs) in paralyzed skeletal muscle. Four‐month‐old male Sprague‐Dawley rats received a moderate‐severe (250 kiloDyne) T‐9 contusion SCI or Sham surgery and were monitored over 2 weeks, and 1‐, 2‐, or 3 months. Animals were sacrificed via isoflurane overdose and terminal exsanguination and the soleus was carefully excised and snap frozen. Protein expression of senescence markers p53, p27, and p16 was determined from whole soleus lysates using Western immunoblotting and RT‐qPCR was used to determine the soleus gene expression of IL‐1α, IL‐1β, IL‐6, CXCL1, and TNFα. SCI soleus muscle displayed 2‐ to 3‐fold higher total p53 protein expression at 2 weeks, and at 1 and 2 months when compared with Sham. p27 expression was stable across all groups and timepoints. p16 protein expression was lower at 3 months in SCI versus Sham, but not earlier timepoints. Gene expression was relatively stable between groups at 2 weeks. There were Surgery x Time interaction effects for IL‐6 and TNFα mRNA expression but not for IL‐1α, IL‐1β, or CXCL1. There were no main effects for time or surgery for IL‐1α, IL‐1β, or CXCL1, but targeted *t* tests showed reductions in IL‐1α and CXCL1 in SCI animals compared to Sham at 3 months and IL‐1β was reduced in SCI animals compared to Sham animals at the 2‐month timepoint. The elevation in p53 does not appear consistent with the induction of SASP because mRNA expression of cytokines associated with senescence was not uniformly upregulated and, in some instances, was downregulated in the early chronic phase of SCI.

## Graphical abstract

Spinal cord injury leads to sustained protein expression of p53 in paralyzed soleus muscles. This change is not associated with differences in key cytokines associated with cellular senescence.

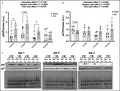

## 1 INTRODUCTION

Spinal cord injury (SCI) is associated with severe reductions in the health of the musculoskeletal system due to disuse and immobilization below the spinal lesion. The extensive muscle atrophy (Qin, Bauman, & Cardozo, 2010) that occurs after SCI is associated with reductions in systemic glucose handling and elevated rates for developing type II diabetes mellitus and cardiovascular diseases (Bauman & Spungen, 2001; Gorgey et al., 2014), outcomes linked with the accumulation of visceral fat tissue (Cirnigliaro et al., 2015) and a potential rise in systemic pro‐inflammatory cytokines (Rosen & Spiegelman, 2014). Skeletal muscle has been recognized to be an endocrine organ (Pedersen & Febbraio, 2012) and it has also been shown to be associated with markers of inflammation in disease states, which cause muscle atrophy (Londhe & Guttridge, 2015), although the exact role of skeletal muscle in creating or sustaining this pro‐inflammatory condition compared to infiltration by immune or other cell types, as well as dysfunctional satellite cells, has not been explicitly answered.

Major changes in the secretome of diseased tissues are common occurrences during cellular senescence. Cellular senescence is a remodeling process in which cells exit the proliferative stage of the cell cycle and signal for immune clearance using the senescence‐associated secretory phenotype (SASP), an event which involves the release of cytokines, chemokines, and other molecules required for cellular breakdown (Munoz‐Espin & Serrano, 2014). Mature skeletal muscle fibers are postmitotic and, thus, lack the ability to proliferate. Accordingly, muscle relies upon the activation, proliferation, and differentiation of resident muscle stem cells called satellite cells during turnover, repair, and regeneration (Rando, 2006). Because muscle atrophy is a major consequence of muscle mass loss after SCI and reduced satellite cell numbers have been reported in individuals with SCI compared to able‐bodied controls (Verdijk et al., 2012), skeletal muscle itself or inflammatory cell infiltration may be responsible for any elevations in SASP. We have shown a complete spinal cord transection results in elevations in pro‐inflammatory cytokine gene expression in paralyzed whole muscle lysates 56 days after SCI compared to Sham controls (Graham, Harlow, & Peng, 2015). This study did not determine which population of cells in the lysate were responsible for the changes in gene expression but does provide some insight that paralysis may affect or induce the expression of genes associated with SASP.

The goal of our study was to determine if muscle disuse led to changes in the time course of pro‐inflammatory cytokine expression associated with SASP across the acute, subacute, and early chronic phases of SCI in rats and to further investigate if protein expression of common senescence markers was similarly changed. Our data show a sustained elevation in total p53 protein expression during the acute and subacute time frame that was not mirrored with greater levels of inflammatory cytokine gene expression, likely suggesting paralyzed skeletal muscle is not associated with a senescent phenotype in the early stages of paralysis.

## 2 MATERIALS AND METHODS

### 2.1 Animals

A subset of left soleus muscles was obtained from a larger experiment evaluating the time course of bone and muscle changes occurring in a rodent moderate–severe contusion SCI model (Otzel, Conover, & Ye, 2018). Briefly, 48 barrier‐raised and specific pathogen‐free 16‐week‐old male Sprague‐Dawley rats were stratified by body mass into 2‐week, 1‐month, 2‐months, or 3‐months group and were randomized into Sham or SCI group that received a thoracic‐level 9 (T_9_) laminectomy with no manipulation of the spinal cord or moderate–severe contusion SCI, respectively (described below). All procedures involving animals conformed to the ILAR Guide to the Care and Use of Experimental Animals and were approved by the Institutional Animal Care and Use Committee at the Malcom Randall VAMC.

### 2.2 SCI, postoperative care, and muscle collection

Surgery and postoperative care were performed per methods previously published by our laboratory (Otzel et al., 2018). Briefly, anesthetized rodents received a T9 laminectomy to expose the spinal cord. A moderate–severe contusion SCI was produced by applying a 250‐kilodyne force to the T‐9 segment of the spinal cord using the Infinite Horizons Impactor (Precision Systems and Instrumentation). Animals received buprenorphine and ketoprofen for 48 hr, and ampicillin for 5 days. Lactated Ringer's saline solution was provided to promote rehydration. Jell‐O® with added protein/fat and apples were provided to assist in maintenance of body mass. Bladders were expressed twice daily until spontaneous voiding returned. Postoperative care included examination for signs of distress, weight loss, dehydration, fecal clearance, bladder dysfunction, and skin lesions. Animals were sacrificed via isoflurane overdose and terminal exsanguination. The soleus was excised, weighed wet, snap frozen in liquid nitrogen, and stored at −80°C.

### 2.3 SDS‐page and western immunoblotting

Methods for SDS‐PAGE and Western immunoblotting have been published previously (Graham, Harlow, & Bauman, 2018). Briefly, for whole muscle lysate analysis, ~30 mg of the left soleus was homogenized by an automated bead homogenizer using a cocktail of 1:10 w/v of 1x RIPA buffer supplemented with phosphatase and protease inhibitors. Muscle homogenates were placed on ice for 10 min and centrifuged at 14,000*g* for 15 min at 4˚C. The supernatant was collected and stored at −80˚C until analysis. Protein content for whole muscle was determined by microBCA using bovine serum albumin (BSA) as the reference standard (Pierce Scientific).

Protein (60 µg) was mixed with 2x Laemmli sample buffer with 5% β‐mercaptoethanol, boiled for 5 min, and run through 4%–20% gradient polyacrylamide gels (Bio‐Rad). Protein was then transferred to a PVDF membrane, stained with Ponceau S, photographed with a CCD digital camera (Amersham Imager 600, GE Amersham), and then destained with a 10 min rinse in Tris‐buffered saline with 0.1% Tween‐20 (TBST). Membranes were then blocked with 5% BSA‐TBST (w/v) for 60 min then incubated overnight at 4˚C with 1% BSA‐TBST buffer containing the appropriate primary antibody. All antibodies were used at a 1:1,000 ratio and the list of antibodies, vendors, and catalog numbers can be found in Table S1. After the overnight incubation, the membranes were placed in TBST for three 10 min washes and then incubated in 1% skim milk/TBST with an HRP‐conjugated secondary antibody (1:2,000 ratio) for 60 min at room temperature. Membranes were washed again in TBST, placed into an HRP‐based chemiluminescent detection solution (ECL Prime; GE Amersham) for 5 min, and then imaged using a CCD digital imager. Densitometry was determined using Image Lab software (Bio‐Rad). Protein expression was normalized using whole‐lane densitometry values after a Ponceau stain (Romero‐Calvo, Ocon, & Martinez‐Moya, 2010).

### 2.4 RNA isolation and RT‐PCR

Total RNA was isolated from 20 mg of the left soleus using a Trizol:chloroform extraction and column centrifugation using the miRNeasy Mini Kit (Qiagen) according to the manufacturer's guidelines. RNA concentrations were measured using a Nanodrop 1000 and 1 µg of sample was used to generate a cDNA library using the RNA using a High‐Capacity RNA‐to‐cDNA kit following the manufacturer's guidelines (Applied Biosystems). cDNA was diluted 1:10 in nuclease‐free water and loaded into 384 well plates with Taqman primers and probes and 2x Taqman Universal PCR Mastermix, both obtained from Applied Biosystems. The list of Taqman gene expression assays and catalog numbers can be found in Table S1. Real‐time PCR was performed using a Quantstudio 12k Flex system and data were compared as relative differences between 18S rRNA and calculated as relative fold change (Livak & Schmittgen, 2001). No fluorescence signal was detected after RT‐qPCR for one Sham animal at the 1‐month timepoint for IL‐1β and IL‐6.

### 2.5 Statistical Analyses

Differences between groups for all parameters were determined using 2 (surgery) x 4 (time) two‐way ANOVA with surgery (Sham and SCI) and time (2 weeks, 1 month, 2 months, and 3 months) being the main effects with multiple comparisons done using Tukey's post hoc test when applicable. Differences between groups were reported when an interaction or main effect had a *p* value < .05. As our major interest was potential outcomes between Sham and SCI groups at each timepoint and not necessarily differences across groups over time, we performed targeted independent samples *t* tests and noted important differences when *p* < .05 between SCI and Sham groups at the same timepoint. Protein and gene expression data were normalized to Sham animals at 2 weeks and are represented as fold change. The results are presented as means ± 95% confidence interval. All statistical values that were calculated are available in Table S2 and were completed using Prism 8 software (Graphpad).

## 3 RESULTS

### 3.1 Muscle mass

**Figure 1:**
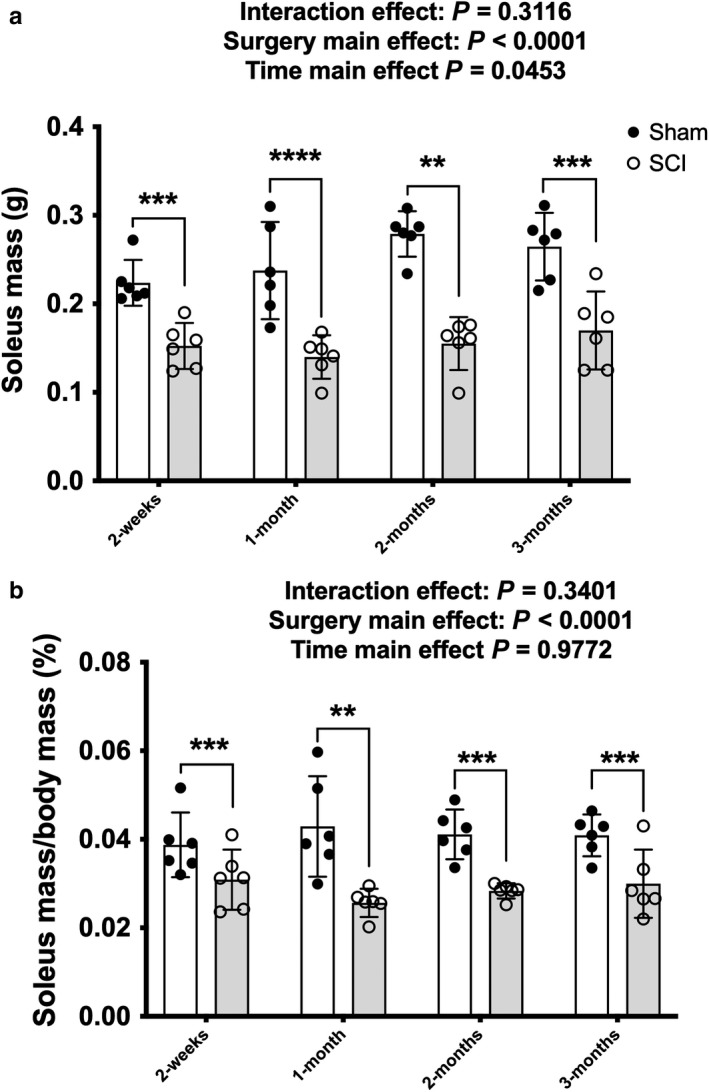
(a) Absolute soleus wet and (b) normalized mass in Sham and contusion SCI animals at 2‐weeks, 1‐, 2‐ and 3‐months post‐injury. Key group differences between Sham and SCI animals are reported at each respective timepoint and ** denotes *p* < .01, *** denotes *p* < .001, **** denotes *p* < .0001. Data are presented as means ± 95% confidence intervals

Main effects for surgery [*F*(1, 40) = 99.98, *p* < .0001] and time [*F*(3, 40) =2.98, *p* = .0453] were present for absolute soleus mass (Figure 1a). A main effect for surgery was also present for soleus mass normalized to body mass [*F*(1, 40) = 44.42, *p* < .0001, Figure 1b]. These findings indicate lower absolute and relative soleus muscle mass in SCI versus Sham animals. Targeted *t* tests indicated that SCI displayed 30% lower absolute soleus mass at 2 weeks (*p* = .003) and 40% lower absolute soleus mass at 1 month (*p* = .001), 2 months (*p* < .0001), and 3 months (*p* = .001) when compared with Sham animals.

### 3.2 Protein expression

**Figure 2:**
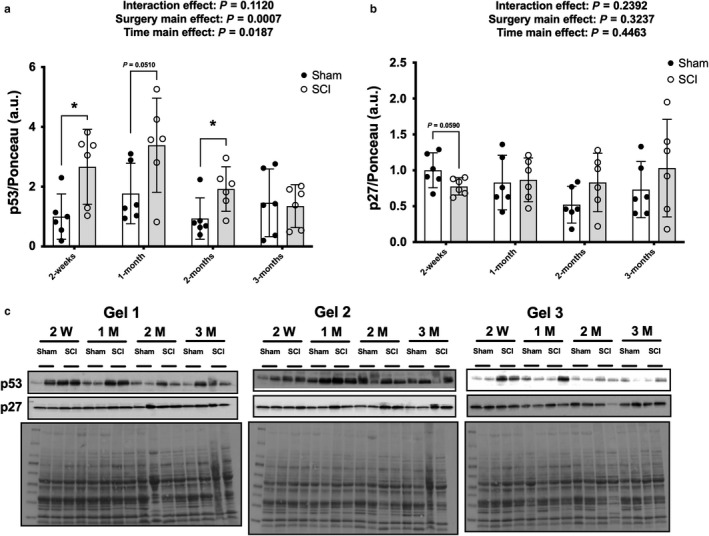
Whole muscle lysate total protein expression of (a) p53 and (b) p27 from the rat soleus at select timepoints after contusion SCI or Sham surgeries. (c) Immunoblots and Ponceau S stains for each individual sample used for the study. Key group differences between Sham and SCI animals are reported at each respective timepoint and * denotes *p* < .05. Data are presented as means ± 95% confidence intervals

**Figure 3:**
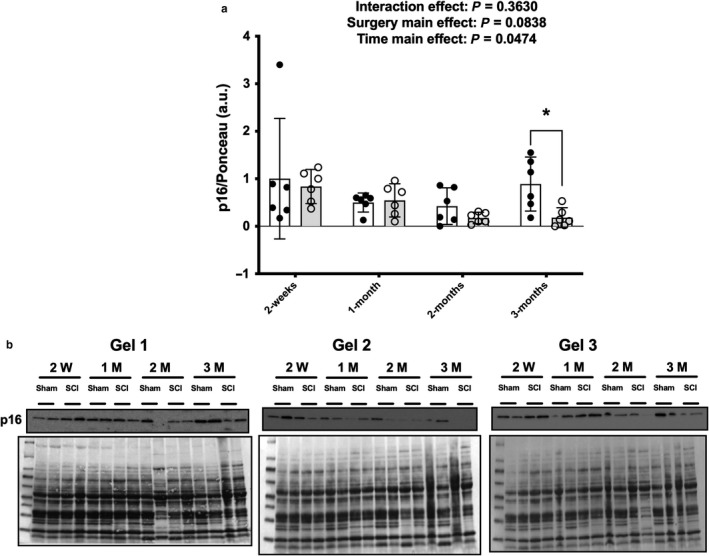
Total protein expression of p16 (a) from the rat soleus at select timepoints after contusion SCI or Sham surgeries. Immunoblots and Ponceau S stains (b) for each individual sample used for the study. Key group differences between Sham and SCI animals are reported at each respective timepoint and * denotes *p* < .05. Data are presented as means ± 95% confidence intervals

There was no surgery x time interaction effect for p53 but there were main effects for surgery [*F*(1, 40) = 13.47, *p* = .0007] and time [*F*(3, 40) = 3.75, *p* = .0187] indicating that SCI displayed higher p53 expression (Figure 2a). Targeted *t* tests indicated elevated p53 protein expression in the SCI groups compared to the Sham group at 2 weeks (*p* = .0152) and 2 months (*p* = .0317), with a strong trend for elevated expression at 1 month (*p* = .0510). There was no interaction or main effect for surgery or time for p27 protein expression (Figure 2b). There was a trend effect for reduced p27 protein expression in the SCI animals at 2 weeks (*p = *.0590) after targeted *t* tests but no further differences at 1‐, 2‐, or 3 months. There was a time main effect [*F*(3, 40) = 2.884, *p* = .04575] for p16 protein expression (Figure 3a) and a targeted *t* test demonstrated that p16 was approximately 80% lower in SCI versus Sham animals at 3 months (*p = *.0134) but no additional differences between groups at the earlier timepoints.

### 3.3 Cytokine mRNA expression

#### 3.3.1 IL‐1α

**Figure 4:**
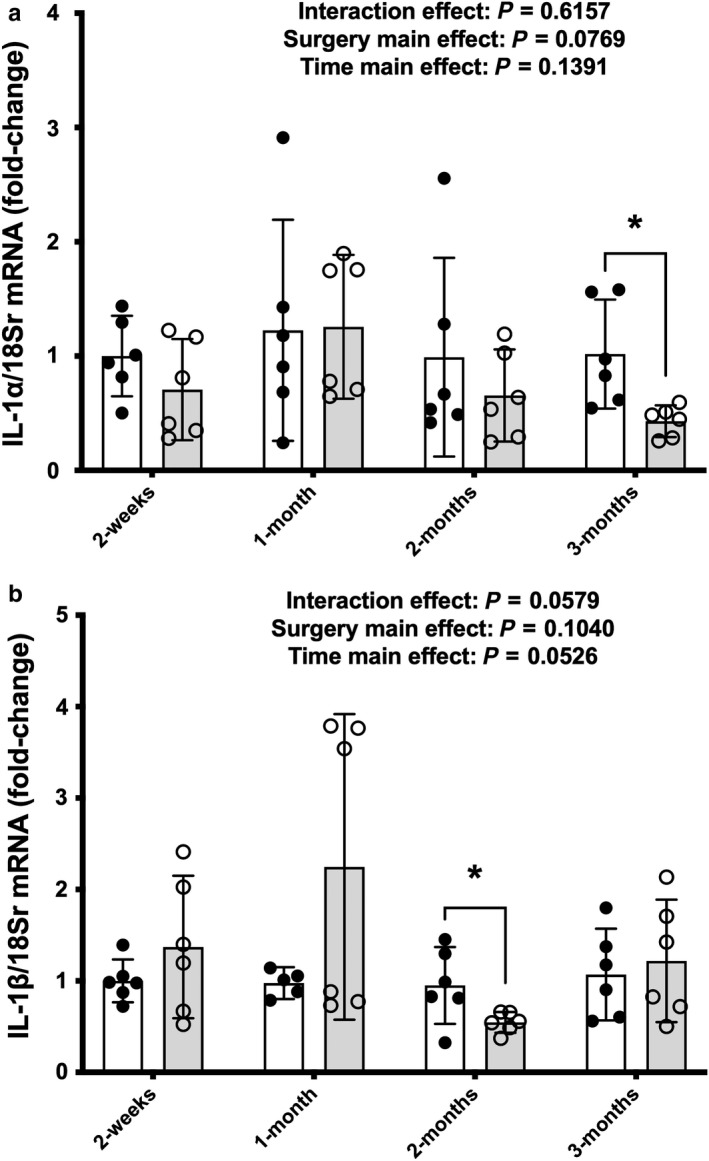
mRNA expression of (a) IL‐1α and (b) IL‐1β from whole soleus muscle at select points across 3 M post‐surgery in Sham or SCI animals. Key group differences between Sham and SCI animals are reported at each respective timepoint and * denotes *p *< .05. Data are presented as means ± 95% confidence intervals

There was no interaction effect or surgery and time main effects for IL‐1α (Figure 4a) but targeted *t* tests showed a reduction in IL‐1α in the SCI group versus Sham animals at 3 months (*p = *.0124).

#### 3.3.2 IL‐1β

There were no surgery x time interaction effect or individual main effect for surgery for IL‐1β mRNA expression (Figure 4b), although there was a trend for a main effect of time [*F*(3, 39, *p* = .053]. Additionally, the 2‐months SCI animals had reduced mRNA expression compared to Sham animals at 2‐months post‐SCI (*p = *.0381) after analysis with targeted *t* tests.

#### 3.3.3 IL‐6

**Figure 5:**
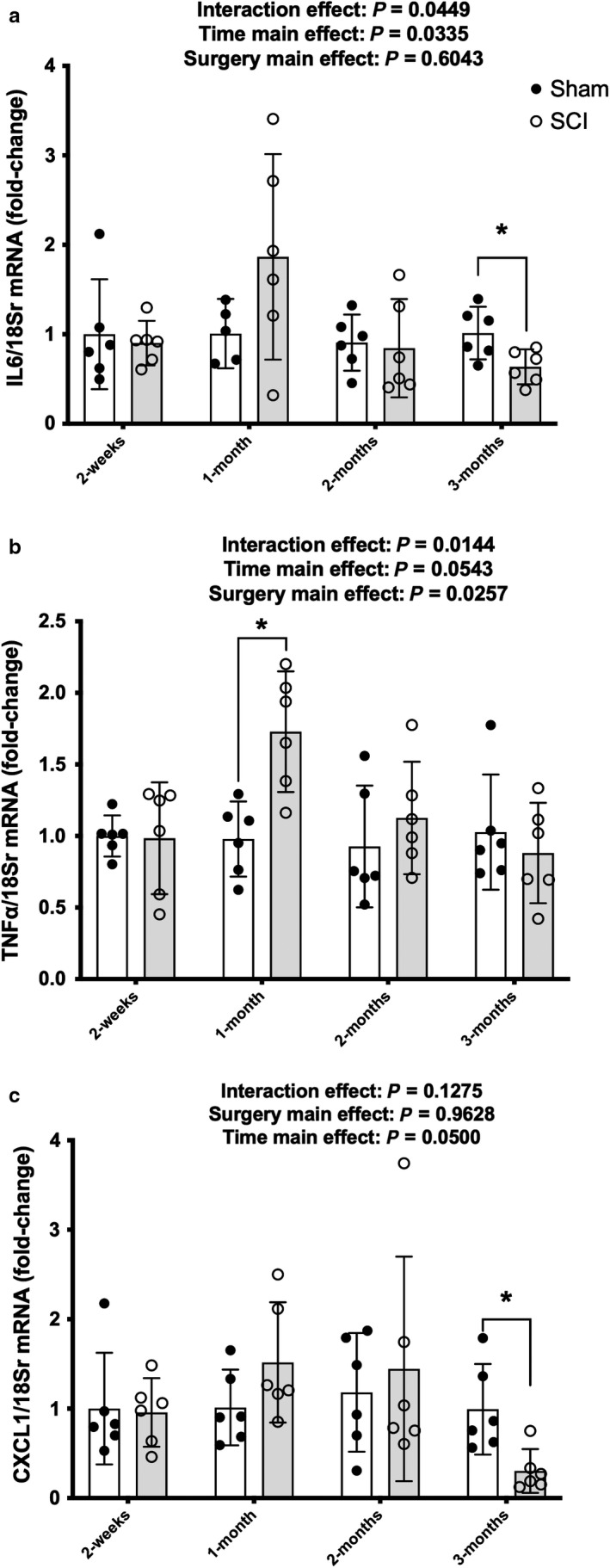
Profile of SASP and atrophy cytokine mRNA expression of cytokines from whole soleus muscle 1 month after Sham or contusion SCI. (a) IL‐6, (b) TNF, and (c) CXCL1. Key group differences between Sham and SCI animals are reported at each respective timepoint and * denotes *p* < .05. Data are presented as means ± 95% confidence intervals

IL‐6 had a surgery x time interaction [Figure 5a; [*F*(3, 39) = 32.941, *p* = .0449]. Follow‐up pairwise comparisons showed the 1‐month SCI group as being elevated compared to 2‐months SCI (*p = *.0331) and 3‐months SCI (*p = *.0052) groups. Targeted *t* tests show that IL‐6 was reduced in the SCI group compared to the Sham group at 3 months (*p = *.0207).

#### 3.3.4 TNFα

There was a surgery x time interaction effect for TNFα mRNA expression [Figure 5b; *F*(3, 40) = 3.974, *p* = .0144]. Follow‐up analyses showed the 1‐month SCI group was elevated compared to the 2‐weeks (*p = *.0147),1‐month (*p = *.0109), 2‐months (*p = *.0052), and 3‐months (*p = *.0211) Sham groups, as well as the 2‐weeks (*p = *.0118) and 3‐months SCI animals (*p = *.0027). *T*argeted *t* tests to determine potential surgical effects between Sham and SCI animals at respective timepoints showed TNFα mRNA expression was elevated in the SCI animals at 1 month (as reported above) but there were no differences between Sham and SCI groups at 2 weeks or 2 and 3 months.

#### 3.3.5 CXCL1

CXCL1 had no interaction or main effects although there was a trend for a main effect of time [Figure 5c; *F*(3, 40, *p* = .0500]. Targeted tests showed relatively stable levels between Sham and SCI groups at 2‐weeks and 1‐ and 2‐months post‐SCI, but a reduction was observed in the SCI group compared to Sham at 3 months (*p = *.0103).

## 4 DISCUSSION

The initial and secondary medical consequences of SCI result in acute and chronic disruption of multiple physiological systems and lead to reductions in the health and quality of life in those injured. The loss of muscle mass is a well‐known characteristic of unloading and paralysis and likely has a role in many of the metabolic disorders associated with SCI. The possible contribution of cell senescence programs to muscle atrophy after SCI is not well understood. We show in a rat model of moderate‐severe contusion SCI that prominent pro‐inflammatory expression of IL‐1α, IL‐1β, and CXCL1 mRNA is relatively stable in skeletal muscle across 2‐months post‐injury despite elevations in p53 protein expression at 2 weeks, 1 month, and 2 months. In comparison, there were interaction effects of IL‐6 and TNFα mRNA that show a transient elevation in expression after SCI and return to baseline, or even reduced levels, at 3‐months post‐injury. These data suggest the rodent soleus, a predominantly slow‐twitch skeletal muscle (Phillips, Beggs, & Ye, 2018), does not reach a state of accelerated cellular senescence during the acute and subacute time frame after moderate–severe contusion SCI.

In our model of contusion SCI, absolute and normalized losses in muscle mass are largely completed by 14 days post‐injury. Acute disuse atrophy is associated with a coordinated cellular program of protein degradation (Bodine, 2013) and changes in the molecular cues for protein synthesis (Gordon, Kelleher, & Kimball, 2013). In a sciatic nerve transection model of disuse atrophy, 3 days of paralysis of the rat soleus muscle resulted in elevated rates of protein degradation as well as elevated rates of protein synthesis using free tyrosine fluorogenic assays (Furuno, Goodman, & Goldberg, 1990). However, protein synthesis as measured by SUnSET is reduced after 14 d of hind limb suspension in the rat soleus (Mirzoev, Tyganov, & Shenkman, 2017) and total fractional synthesis rates of the soleus muscle are reduced after 28 d of unloading, while no change in protein synthetic rate is observed for the myofibrillar fraction (Shimkus, Shirazi‐Fard, & Wiggs, 2018). While these different models of disuse atrophy have distinct molecular responses, it is likely that the initial loss in skeletal muscle mass in rats is predominantly due to increases in degradation rates, which are largely completed by 14 d. The minimal changes in muscle mass observed thereafter are due to blunted protein synthesis rates in the absence of reloading.

In skeletal muscle, studies of cell senescence have been largely focused on dysfunctional satellite cells and their effect on muscle health during normal aging and sarcopenia (Sousa‐Victor & Munoz‐Canoves, 2016), with a gradual accumulation of point mutations being associated with reductions in satellite cell function (Franco, Johansson, & Olsson, 2018). Poorly functioning satellite cells would likely be detrimental to local muscle fiber health and impact regeneration and recovery after an injury. SCI has been associated with reduced satellite cell numbers in humans (Verdijk et al., 2012) and reduced myonuclear numbers 8 weeks after complete transection in rats (Dupont‐Versteegden, Murphy, Houle, Gurley, & Peterson, 2000). However, satellite cells isolated from individuals with chronic SCI have similar characteristics to able‐bodied controls in the proliferative myoblast stage and during differentiation (Savikj, Ruby, & Kostovski, 2018). Thus, it is likely that any appreciable change in skeletal muscle cytokine levels is not coming from satellite cells after paralysis due to SCI.

Skeletal muscle inflammation has been investigated in many pathological states, as well as after traumatic injuries such as burns and exercise‐induced muscle damage. Elevated muscle inflammation is associated with aging, and primary cells isolated and cultured from vastus lateralis biopsies of aged individuals show greater downstream NF‐κB activation response to recombinant TNFα as well as blunted differentiation and fusion compared to young individuals (Merritt, Stec, & Thalacker‐Mercer, 2013). A complete spinal cord transection in female mice 8 weeks after the injury resulted in greater IL‐1β, IL‐6, and TNFα mRNA expression in paralyzed gastrocnemius muscles (Graham et al., 2015). Chronic SCI has also resulted in greater vastus lateralis expression of the pro‐atrophy inflammatory regulators Fn14 (TWEAK receptor) and TNFα receptor 1B in humans, although there were no changes in the mRNA expression of IL‐6, TNFα, or TWEAK. These elevations were seen together with greater Fn14 and activated NF‐κB‐p65 protein expression and further suggest greater inflammation sensitivity in paralyzed muscle (Yarar‐Fisher, Bickel, & Kelly, 2016). Our current data conflicts with our previous study in mice in which we reported an upregulation of pro‐inflammatory cytokines in the gastrocnemius of paralyzed mice following a complete spinal cord transection (Graham et al., 2015). The differences in effects of the SCI on expression of pro‐inflammatory cytokines between these studies may be explained by the difference between a spinal cord transection, which completely severs communication between the peripheral and central nervous systems below the area of injury, compared to a moderate–severe contusion injury, which may leave residual connections that can regulate muscle despite near complete paralysis of the hind limbs. Alternatively, it is possible that selection of different muscles (i.e., soleus vs. gastrocnemius) may explain differences between our current and previous findings, given that the soleus is predominantly slow‐twitch in rat and the mouse gastrocnemius is a largely glycolytic muscle. In this regard, others have reported that IL‐6 protein is released from soleus but not the predominantly fast‐glycolytic extensor digitorum longus (EDL) of mice, using an ex vivo assay, and that IL‐6 protein expression was primarily expressed in small oxidative fibers of the gastrocnemius and not in larger glycolytic fibers (Liang, Drazick, Gao, & Li, 2018). Furthermore, it should be noted that a profound slow‐oxidative to fast‐glycolytic fiber‐type shift occurs in the soleus in our moderate–severe contusion SCI model within the time frame of our current study (Yarrow, Kok, & Phillips, 2019) and it remains unclear whether this phenotypic change has any influence on our findings.

The cytokines associated with SASP, such as the early secretion of IL‐1α and the later elevations of IL‐6 and CXCL1/GRO, would be expected as they are a characteristic of the development of senescence (Rodier & Campisi, 2011). However, levels of expression of these cytokines at 2‐weeks and at 1‐ and 2‐months post‐injury in the present study were either unchanged or modestly and variably altered, with the exception of a marked reduction in IL‐6 and CXCL1 at 3‐months post‐SCI. The reasons for this are unclear but may be related to the reduction in p16 protein expression at the same timepoint. p16 is expressed by most senescent cells (Munoz‐Espin & Serrano, 2014) and reductions in p16 levels using genetic knockouts have been demonstrated to reduce levels of both mRNA and protein levels of IL‐1β, IL‐6, and TNFα in a renal model of stress‐induced premature senescence (Jin, Tao, & Gu, 2017). The sustained elevation in total p53 protein expression without any drastic change in cytokine mRNA levels in our SCI animals suggests there may be ancillary pathways involved which may limit the effect of constant elevations in p53 on cytokine expression; alternatively, at least in muscle, p53 may be playing an important role in muscle atrophy, with this latter suggestion being the more likely explanation. For example, p53 has been shown to be a prominent muscle atrophy factor. p53 is elevated in skeletal muscle during immobilization in wild‐type mice but in mice with a muscle‐specific knockout of p53, muscle atrophy was greatly reduced after immobilization (Fox, Ebert, & Bongers, 2014). Additionally, muscle atrophy occurs following plasmid‐induced overexpression of p53 and not with a mutant p53 vector (Fox et al., 2014). Interestingly, the highest relative p53 expression was observed at 2‐weeks and 1‐month post‐SCI, which represented the timeframe in which nearly all muscle loss occurred in our experimental rodent model. While we note elevated p53 in SCI compared to Sham animals at 2 months but no difference at 3 months, others have found p53 to be downregulated 10 weeks after a complete SCI in rats (Fry, Drummond, Lujan, DiCarlo, & Rasmussen, 2012).

Our data show a rapid increase in p53 protein expression that is sustained for the first 2 months post‐contusion SCI in rat soleus muscle. This was not associated with drastic increases in mRNA expression of cytokines commonly secreted by cells with a SASP profile. At 3‐months post‐SCI, when skeletal muscle atrophy has essentially stopped, p16 protein expression was reduced in conjunction with a reduction in the expression of mRNA encoding several key cytokines that are associated with cellular senescence. Our studies are limited by the use of whole muscle homogenates, which contain multiple cell types in normal healthy muscle and are further infiltrated by additional cell types after paralysis and resulting disuse. Additionally, the measurements of these cytokines in collaboration with mechanistic studies using transgenic animals or targeted drugs could provide additional information regarding SCI, especially at the early timepoints when muscle loss is most rapid. Single fiber secretome studies of chronically paralyzed skeletal muscle would provide much needed clarity on the degree of the role played, if any, of muscle SASP and cell senescence in driving muscle atrophy; furthermore, a single fiber approach to this question would assist in clarifying whether the phenotypic shift from slow‐to‐fast fiber‐type that occurs after SCI alters the rate or magnitude of muscle loss.

## CONFLICT OF INTEREST

The authors report no conflict of interest.
